# Localized Sound‐Integrated Display Speaker Using Crosstalk‐Free Piezoelectric Vibration Array

**DOI:** 10.1002/advs.202414691

**Published:** 2025-04-25

**Authors:** Inpyo Hong, Su Seok Choi

**Affiliations:** ^1^ Graduate School of Semiconductor Technology Pohang University of Science and Technology (POSTECH) Pohang 37673 Republic of Korea; ^2^ Graduate School of Electrical Engineering Pohang University of Science and Technology (POSTECH) Pohang 37673 Republic of Korea

**Keywords:** display speaker, flat panel speaker, localized vibration, multisensory display, piezoelectric speaker

## Abstract

Beyond visual quality, features like sound and tactile feedback have become essential to enhancing user experience, resulting in more immersive, realistic displays. Multisensory displays engaging multiple senses are increasingly in demand. Panel‐integrated piezoelectric speakers represent a major advancement in audio‐visual technology, merging sound generation with display panels to enable compact, versatile designs in thin, flexible formats. However, challenges like sound crosstalk between exciters and non‐uniform frequency responses often compromise audio quality. To address these issues, frame‐based sound vibration isolation strategies are explored to localize surface vibrations and reduce interference across multiple exciters. Through experimental measurements and Finite Element Method (FEM) simulations, it is found that increasing frame height and width, along with using materials with different acoustic impedance for the diaphragm, significantly improved frequency response uniformity and reduced Total Harmonic Distortion (THD). These enhancements simplify speaker response compensation, ensuring reliable, high‐quality sound output. As demonstrated on a practical 13‐inch OLED display, these results confirm that vibration‐isolated, localized sound in multi‐array exciters overcomes prior limitations, advancing the acoustic performance of piezoelectric panel speakers. This study provides valuable insights for future developments in thin, flexible, display‐integrated audio systems, offering new possibilities in immersive, multisensory user experiences.

## Graphical abstract

To approach multisensory integration of speaker‐integrated OLED displays, this study investigates localized sound emission in display panel speakers by examining crosstalk‐free diaphragm vibrations and enhanced frequency response through multi‐arrayed piezoelectric exciters. The objective is to minimize sound vibration interference among multiple exciters and achieve a uniform frequency response. By optimizing a vibration‐isolating frame structure, vibration confinement is improved, reduced Total Harmonic Distortion (THD), and enhanced sound clarity. Long‐term tests on a 13‐inch OLED display confirms stable performance, highlighting the potential of frame‐based designs in advanced audio‐visual systems.

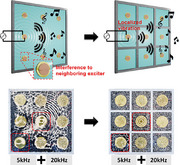

## 1 Introduction

The dominance of sight and hearing among human senses has driven advancements in both display and audio technology, with recent interest in multisensory integration for more immersive experiences.^[^ ^1^, ^2^, ^3^ ^]^ Therefore, beyond the focus on visual quality in displays until now, features like sound and tactile feedback have become essential for enhancing user experience, resulting in more immersive and realistic displays. So‐called, “Multisensory Displays”, combining visuals with sound and touch, are becoming essential for enhancing user engagement and realism.^[^ ^4^, ^5^, ^6^, ^7^ ^]^ To develop this multisensory display, additional functions such as sound and touch have become increasingly important, alongside the visual quality of screens, for enhancing the overall user experience and contributing to a more immersive and realistic display. In short, a multisensory display that engages multiple human senses to complement visual information is highly desirable.

As a step toward multisensory integration, acoustic transducers are increasingly being developed to expand functionality beyond sound generation, with recent studies exploring their application as tactile sensors^[^ ^8^, ^9^, ^10^, ^11^ ^]^ and actuators.^[^ ^12^, ^13^, ^14^, ^15^, ^16^, ^17^ ^]^ In psychoacoustics, aligning auditory and visual information is essential for accurate perception of external stimuli.^[^ ^18^ ^]^ However, in display devices like TVs, smartphones, and automotive, the integration of speakers often conflicts with the slim form factor, and the spatial mismatch between the perceived sound source and actual speaker location can reduce immersion. Addressing these challenges is key to advancing multisensory display experiences.

Various panel speaker technologies have been explored to integrate sound generation directly into displays.^[^ ^19^, ^20^, ^21^, ^22^ ^]^ Electrostatic speakers, for example, have a simple structure but suffer from high power consumption and poor sound quality.^[^ ^23^, ^24^, ^25^ ^]^ Thermoacoustic speakers, which generate sound through the rapid heating and cooling of conductive materials, offer advantages in thinness and compactness but struggle with low sound pressure levels and limited compatibility with electronic devices.^[^ ^26^ ^]^ While these technologies highlight the potential for display‐integrated speakers, they also present challenges in terms of efficiency, performance, and practicality.^[^ ^27^, ^28^, ^29^, ^30^ ^]^ This preferred approach utilizes OLED's ultra‐thin, self‐emissive structure to enable seamless acoustic integration, in contrast, numerous layers and rigid components of LCD and mini‐LED panels hinder efficient vibration transmission, making them unsuitable for diaphragm‐based panel speaker applications.^[^ ^31^ ^]^

Among commercially implemented panel speakers, LG Display's Crystal Sound OLED (CSO) employs electromagnetic speakers, where enclosure tape is used to suppress unwanted vibrations.^[^ ^31^, ^32^ ^]^ However, electromagnetic speakers inherently require coils, leading to a thicker structure that is not ideal for modern ultra‐thin and flexible displays.^[^ ^33^, ^34^, ^35^, ^36^ ^]^ Similarly, Sony's Acoustic Surface Audio integrates speakers into the display. These commercialized products demonstrate the feasibility of panel speakers but also highlight the structural limitations of electromagnetic speakers, emphasizing the need for alternative solutions that align better with evolving display technology trends.

To overcome these limitations, piezoelectric speakers have emerged as a promising alternative.^[^ ^37^, ^38^ ^]^ Piezoelectric speakers directly convert electrical energy into mechanical motion via the inverse piezoelectric effect, enabling efficient sound generation with a lightweight, flexible, and low‐power design. Their simple layered construction, consisting of electrodes and piezoelectric materials, offers advantages such as low cost, high energy efficiency, and compactness.^[^ ^20^, ^39^, ^40^, ^41^, ^42^ ^]^ Additionally, materials like polyvinylidene fluoride (PVDF) allow for flexible, large‐area speaker applications, making them highly suitable for integration with next‐generation displays.^[^ ^43^, ^44^, ^45^ ^]^ While various piezoelectric technologies are under development, most studies focus on a single element (or “exciter”) rather than addressing multi‐element configurations. In practice, however, speakers often require multiple exciters arranged in arrays to improve performance and achieve realistic stereo effects. Despite this need, there has been limited research into the challenges of driving multiple exciters independently and resolving system‐wide issues. This contrasts with localized image control techniques in displays, such as high dynamic range (HDR) and local brightness dimming control, which maximize visual realism.^[^ ^46^, ^47^ ^]^ Furthermore, previous studies have primarily focused on optimizing material properties and structural configurations for acoustic performance, while neglecting vibration control through impedance discontinuities, expressed as $Z=\sqrt{\rho \cdot E}$ (ρ and *E* are density and Young's modulus respectively). This gap in research highlights the need for alternative methods to achieve precise diaphragm vibration control, particularly in multi‐element piezoelectric speaker systems.

Here, this study addresses two critical aspects of piezoelectric panel speakers: achieving crosstalk‐free diaphragm vibrations and enhancing consistency of frequency response through localized control of multi‐arrayed piezoelectric exciters. Panel speakers exhibit complex vibrations on the diaphragm surface, which can degrade sound quality when different signals are applied to exciters sharing a single diaphragm in a stereo system, leading to interference. Maintaining a consistent frequency response across exciters is particularly important, as human auditory perception is highly sensitive to sound pressure level (SPL) variations, especially in the mid‐range frequency band, where inconsistencies can be more perceptible and disruptive. However, in multi‐exciter configurations, these inconsistencies are often unavoidable, making compensation even more complex. Typically, response compensation in speakers involves crossovers, filters, or digital signal processing (DSP), with demands increasing alongside the number of speaker channels. In panel speakers, where each exciter has a different frequency response, the demand for compensation becomes even more significant.

To address these issues, we introduced vibration‐isolating frames made from materials stiffer than the diaphragm, such as the display panel, to separate the regions between exciters. This design confines vibrations to the targeted area of each exciter, effectively preventing interference. Unlike conventional studies focusing on high SPL and low total harmonic distortion (THD), this research is not aimed at improving absolute speaker performance but rather focuses on vibration localization and frequency response consistency through structural modifications. Using Chladni patterns, we verified crosstalk‐free vibrations on the diaphragm surface, confirming the frames’ effectiveness in isolating exciters. Without frames, the diaphragm shows variable frequency responses based on exciter location; with frames, each exciter operates within its own defined section, achieving localized vibration and consistent frequency response. This frequency response uniformity simplifies compensation processes, reducing the need for complex DSP, crossover, and filtering adjustments. As a result, a 13‐inch OLED panel with crosstalk‐free piezoelectric speakers was demonstrated, achieving enhanced frequency response through localized control of multi‐arrayed exciters. Beyond vibration isolation, this study advances system‐level speaker array design by integrating multiple vibrating elements. Unlike prior research that separately addresses material optimization (SPL, THD) or signal‐based compensation (circuit, algorithm design), our approach bridges these domains. By reducing frequency response deviation and THD at the structural level, it enables more efficient post‐processing, minimizing computational overhead and algorithmic corrections in multi‐channel setups. This physics‐driven, material‐aware optimization offers a scalable, integrated solution for next‐generation display and audio technologies.

## 2 Results and Discussion

### 2.1 Vibration Isolation for a Sound Crosstalk‐Free Speaker Exciter Array

**Figure 1:**
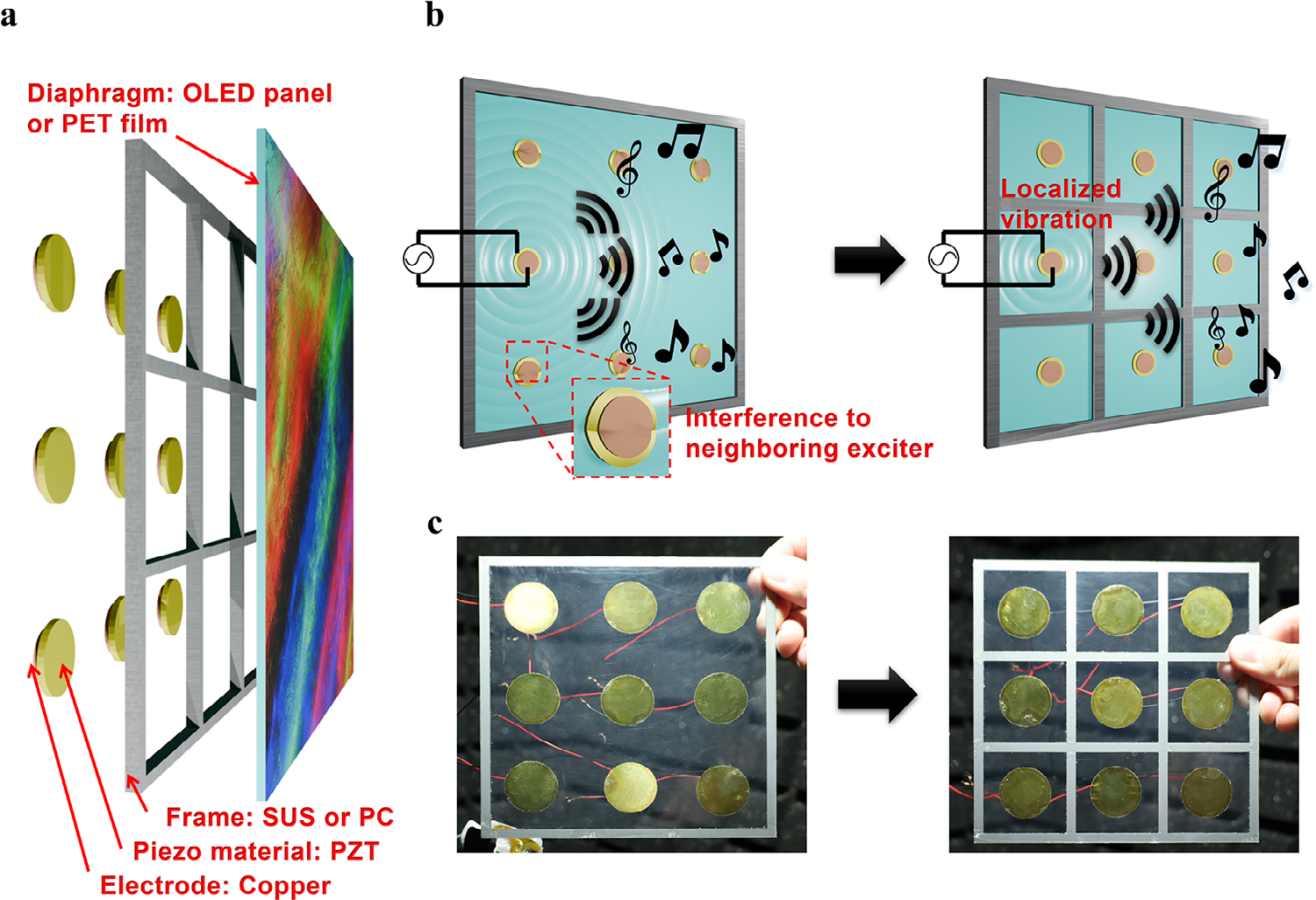
Concept of a vibration‐localized piezoelectric panel speaker. a) Configuration of localized sound emitting flat panel piezoelectric loudspeaker. b) Illustration showing the vibration of the diaphragm and the generation of sound when a sound signal is applied to one exciter in a model with a frame only on the outer edges and in a model where each region of the exciters is separated by a frame. c) Picture of two piezoelectric speaker models: without and with an inner frame of isolating sound vibration.

The panel‐type piezoelectric speaker consists of multiple exciters sharing a single diaphragm such as an OLED display panel. In this configuration, each exciter generates vibrations through the same diaphragm, leading to potential vibration interference between exciters that can degrade sound quality, especially in stereo systems. Additionally, the position of the exciters in a panel speaker significantly impacts the frequency response, resulting in variations in frequency response among the exciters array. **Figure** **1** illustrates the structure of the piezoelectric panel speaker used in this research. The panel speaker was constructed by attaching commercially available lead zirconate titanate (PZT) elements to the diaphragm, as shown in Figure 1a. Each PZT element consists of a three‐layer structure: a bottom electrode, a PZT material, and a top electrode. Frames were added to this speaker structure, with one model supporting only the outer edges and another featuring frames that isolate each exciter's region to localize vibrations of sound. The wave generated by a signal applied to a single exciter propagates across the diaphragm, resulting in surface vibrations (Figure 1b). While these vibrations typically spread across the diaphragm, the frame design confines each exciter's vibrations to its designated area, reducing interference and distortion. Additionally, positioning each exciter centrally within its diaphragm section promotes a uniform frequency response across the array. In this research, with a better understanding of the mechanism to achieve localized vibration and improve sound quality, we simulated and fabricated a speaker with frame‐separated regions for each exciter. This design demonstrates a significant enhancement in sound quality by minimizing exciter interference and ensuring a consistent frequency response. Detailed dimensions of the speaker are provided in Figure 1c, with additional images available in Figure S1 (Supporting Information).

**Figure 2:**
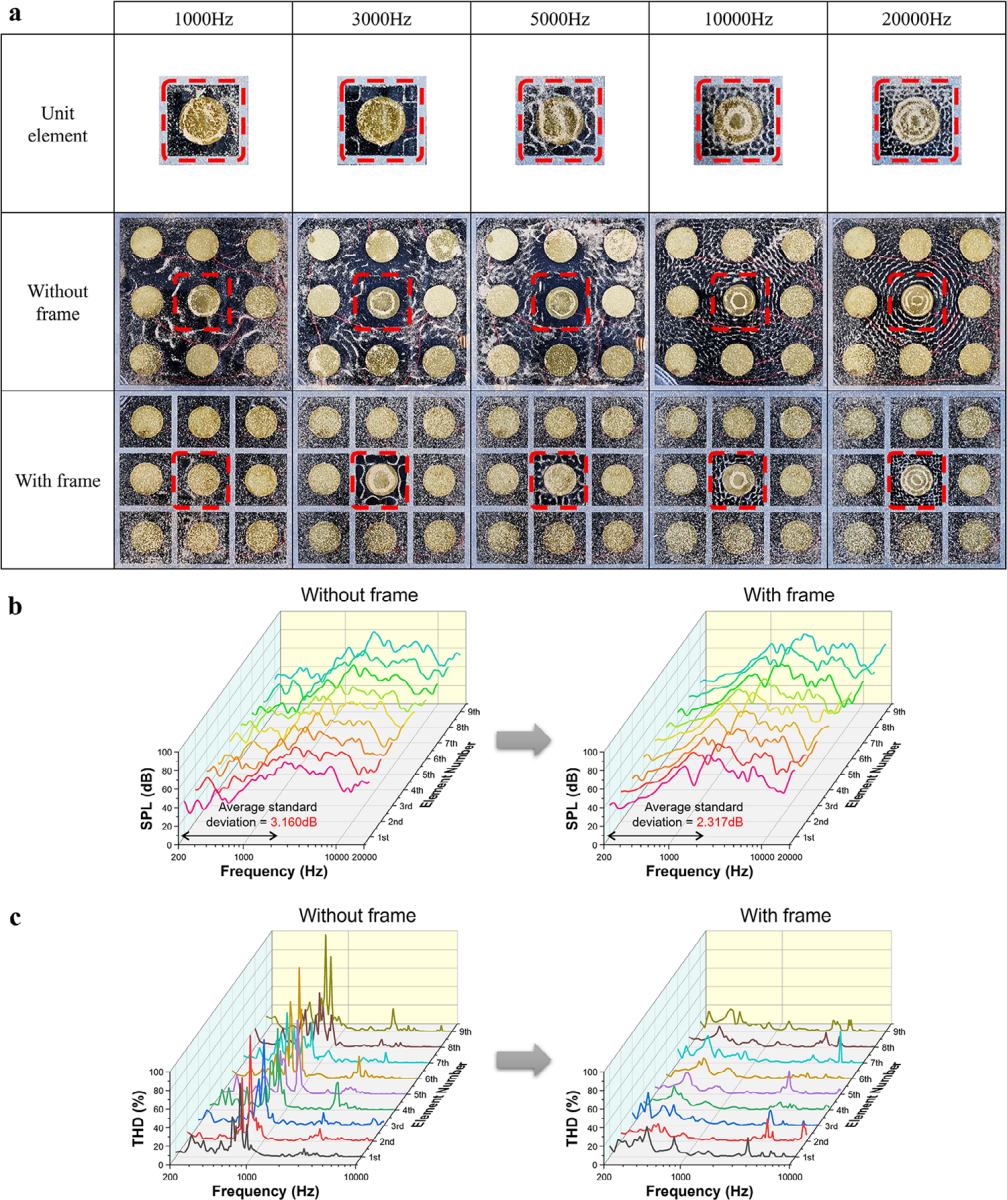
Speaker response with and without a vibration isolating frame. a) Chladni patterns of the unit element, without frame model, and with frame model at various frequencies. b) On‐axis responses of 9 exciters. c) THD of the exciter array sharing a single diaphragm, comparing the cases with and without a frame for various positions of the signal‐applied element.

The performance of a speaker can be evaluated through various factors. In this study, we measured the speaker's frequency response, Chladni patterns (visualizing surface vibrations using fine particles like sand), and THD as shown in **Figure** **2**. Detailed experimental setup for frequency response and THD measurements are provided in Figure S2 (Supporting Information). To assess the effect of vibration‐isolating frames that separate exciter regions, we compared the speaker's response with and without frames. We confirmed that implementing localized vibration through frames did not impact time stability, voltage linearity, or distance response.

Figure 2a illustrates the Chladni patterns for three different panel speaker models to observe surface vibration propagation from a centrally located exciter. Chladni patterns at various frequencies are shown for three models: a single piezoelectric exciter(unit element), a model with a 3×3 array of piezoelectric elements on a single diaphragm with only the outer edges rigidly fixed, and a model with frame‐separated regions. The pattern indicates that in the model without divided regions, the vibrations from the central exciter propagate into surrounding areas. Figure 2b presents the on‐axis frequency response for each exciter location, with each element numbered starting from the top‐left of the 3×3 arrays. In the model without divided regions, the frequency responses vary across exciters. Consistent exciter performance is essential for high‐quality sound unless specific frequency bands are intentionally emphasized. Consistent exciter performance simplifies the signal processing required for frequency response compensation using crossovers, filters, and equalizers. Figure 2c presents the THD across different positions for both models. THD can be expressed by the following Equation (1).

(1) $$THD=\frac{\sqrt{\sum_{n=2}^{N}P_{nth\;harmonic}^{2}}}{P_{1}}*100$$

where *P*_1_ *and* *P_n_* are root mean square (RMS) sound pressure of the first and *n*th harmonics, respectively. In the model without frame separation, the frequency with high THD varied depending on their position, and the overall THD values were higher. However, frame‐separated model, the overall THD was relatively lower. Additionally, even if the exciter's position changes, the frequency bands where THD increases remain similar. Lower THD indicates accurate signal reproduction, yielding clearer sound. Similar to frequency response, THD can be compensated through signal processing. Thus, separating exciter regions with frames not only reduces THD but also aligns high‐THD frequency bands, simplifying the signal processing required for sound compensation.

### 2.2 The Effect of Vibration‐Isolating Frame Shape Structure on Speaker Performance

The wave generated on the diaphragm of a panel speaker by an excited piezoelectric element is absorbed by the frame and transmitted to the neighboring exciter's region. When simplified to one dimension, the wave equation for the frame area is given by Equation (2)^[^ ^48^ ^]^:

(2) $$\frac{\partial^{2}y}{\partial t^{2}}+2\beta \frac{\partial y}{\partial t}-\nu_{1}\;\frac{\partial^{2}y}{\partial x^{2}}=0$$

where *y* is the amplitude of the wave, *t* is time, β is the temporal absorption coefficient, ν_1_ is the speed of the wave, and *x* is the propagation direction. The coefficient β is determined by the material properties and, in this research, corresponds to the material of the frame. Even with the same material, the dimension of the frame also affects the coefficient, with larger dimensions generally resulting in a higher coefficient.

Numerous studies have shown that the absorption coefficient of acoustic waves is influenced by frequency and attenuation. Equation (3) describes the relationship between frequency and the absorption coefficient, while Equation (4) represents the relationship between pressure and distance.^[^ ^48^ ^]^

(3) $$\beta \;\left(\omega \right)=\beta \omega^{\eta }$$

(4) $$P\;\left(x+\Delta x\right)=P\left(x\right)e^{-\beta \left(\omega \right)\Delta x}$$

where ω is the angular frequency, η is a non‐negative material parameter, *P* is pressure, *x* is position, and Δ*x* is the wave propagation distance. Equation (4) shows that the pressure of the wave decreases exponentially as the propagation distance increases.

The attenuation of diaphragm vibrations due to the frame also occurs as a result of acoustic impedance discontinuity at the boundary when a wave propagates through materials with different impedances. The energy reflection R and transmission T coefficients can be expressed as:

(5) $$R={\left(\frac{Z_{B}-Z_{A}}{Z_{B}+Z_{A}}\right)}^{2}$$

(6) $$T=\frac{4Z_{A}\times Z_{B}}{{\left(Z_{B}+Z_{A}\right)}^{2}}$$

where *Z_A_* and *Z_B_* are the acoustic impedances of the diaphragm and frame, respectively.

We investigated the effect of the acoustic impedance of frame materials on surface wave propagation by analyzing speaker performance. The diaphragm, made of PET, has an acoustic impedance of 3.2 × 10^6^ *kg*/*m* ^2^ · *s*, while the frames were composed of polycarbonate (*Z_PC_* =  2.1 × 10^6^ *kg*/*m* ^2^ · *s*), representing a lower impedance difference, and stainless steel (*Z_SUS_* =  39.0 × 10^6^ *kg*/*m* ^2^ · *s*), representing a higher impedance difference. Substituting these values into Equations (5) and (6) yields reflection and transmission coefficients highlighting the impact of impedance mismatch on wave propagation. Specifically, for the PET diaphragm and SUS frame, the calculations result in R = 72.3% and T = 27.7%, indicating significant reflection at the boundary. In contrast, for the PET diaphragm and PC frame, the results yield R = 4.2% and T = 95.8%, demonstrating a much higher degree of wave transmission. These findings confirm that higher acoustic impedance contrast leads to greater wave reflection, effectively confining vibrations within the designated exciter regions, while lower contrast allows for greater wave transmission across the diaphragm.

To examine how vibration attenuation due to impedance mismatch influences interference between exciters, we attached vibration‐isolating frames made of materials with both low and high impedance differences to a panel speaker with a 3×3 array of piezoelectric elements.

**Figure 3:**
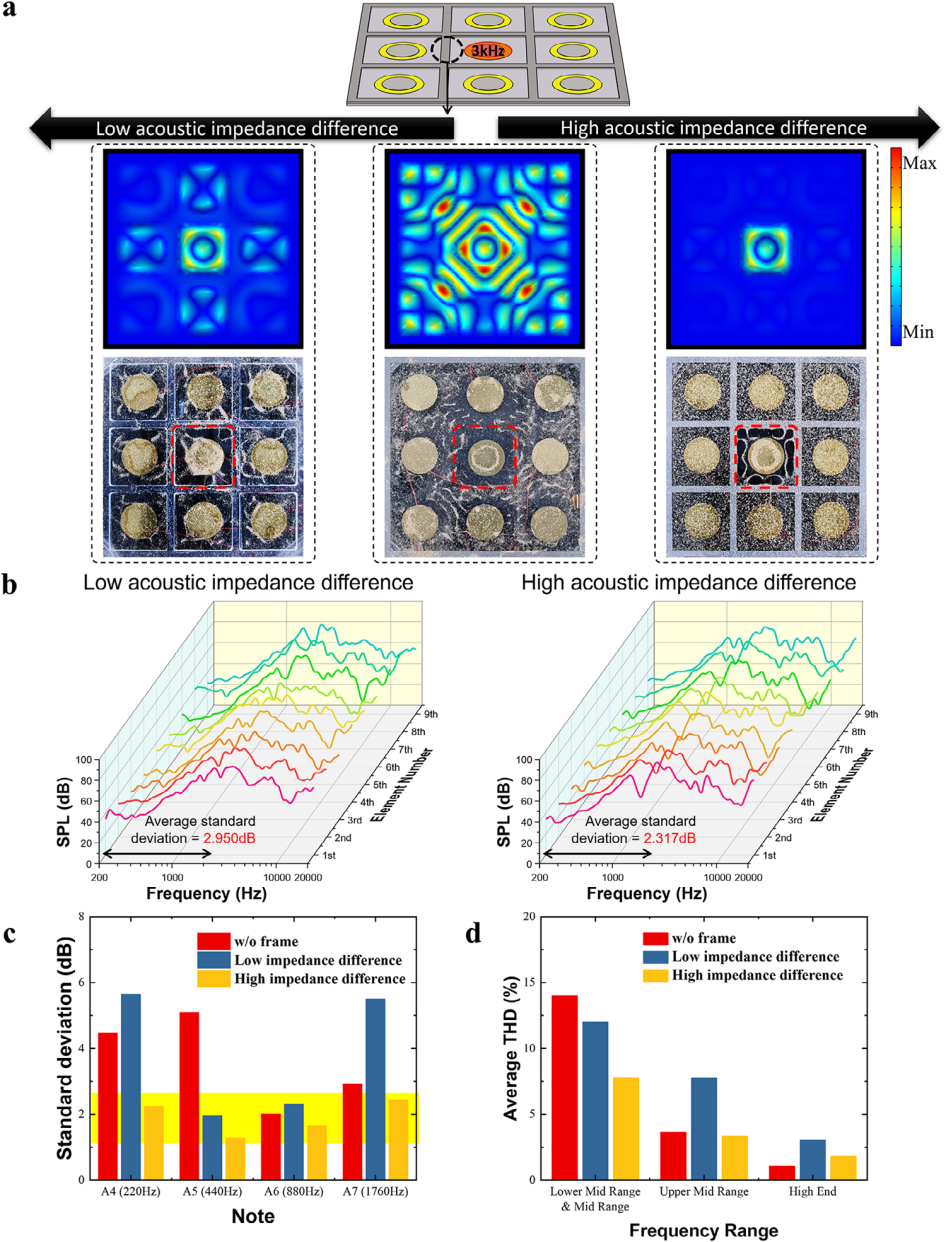
Speaker characteristics are determined by the acoustic impedance difference between the frame and the diaphragm. a) FEM results, and Chladni patterns showing surface vibration of the diaphragm when a signal is applied to the central element. b) The frequency response of the panel speaker. c) SPL deviation of A notes produced by 9 exciters in the piezoelectric element array of the speaker. d) Average THD of sound produced by 9 exciters in the piezoelectric element array of the speaker across the frequency bands used in audio engineering.

When a signal was applied to the central exciter, we examined diaphragm vibrations using Chladni patterns and Finite Element Method (FEM) simulations (**Figure** **3**a). At 3 kHz, the frame with a small impedance difference exhibited improved vibration localization compared to the no‐frame condition but still transmitted significant vibrations to neighboring exciters. In contrast, the high‐impedance difference frame confined vibrations more effectively to designated regions, and this trend was observed across other frequencies as well (Figures S3 and S4, Supporting Information). Additionally, the Chladni patterns were measured for three types of speakers: no frame, regions divided by SUS, and regions divided by PC, with a 10Vpp sine wave swept from 20 to 20000 Hz over 30 s for each speaker (Video S1, Supporting Information). The acoustic impedance of the frame also influenced the deviation in frequency response between the exciters (Figure 3b). The speaker with the small impedance difference frame exhibited an average standard deviation of 2.950 dB among exciters within the 200–2000 Hz range, while the speaker with the large impedance difference frame showed a lower average standard deviation of 2.317 dB, indicating greater frequency response consistency among the exciters. Figure S5a (Supporting Information) displays a plot overlaying the frequency responses among the exciters. When examining the standard deviation at frequencies corresponding to the A notes, the small impedance difference frame exhibited higher deviations at certain notes, whereas the large impedance difference frame maintained consistently low deviation levels (Figure 3c; Figure S5b, Supporting Information).

From the perspective of THD, which reflects sound clarity, an improvement was also observed with the frame (Figure 3d; Figure S5c, Supporting Information). While the small impedance difference frame showed THD improvement in lower‐ mid and mid‐range frequencies, it resulted in increased THD in the upper mid‐range and high‐end frequencies. In contrast, the large impedance difference frame consistently reduced THD across all frequency ranges.

Using frame materials with varying acoustic impedance values, we observed through FEM simulations that as the impedance mismatch between the diaphragm and frame increased, vibrations transmitted to neighboring regions decreased significantly. Figure S6 (Supporting Information) presents the reflection and transmission ratios calculated using Equations (5) and (6) for a PET diaphragm and each frame material. As shown in the results, the SUS frame, which has the highest acoustic impedance difference with the PET diaphragm, exhibits the lowest wave transmission, confirming the significant effect of impedance mismatch on vibration localization.

### 2.3 Impact of Vibration Isolating Frame Dimension on Speaker Response

#### 2.3.1 Variation of Speaker Response with Frame Width

In this study, we observed that waves generated by the central piezoelectric element propagate into neighboring regions, with attenuation depending on the frame width along the propagation path. This suggests that the width of the sound vibration‐isolating frame plays a critical role in confining surface vibrations within the region of each exciter on the piezoelectric panel speaker.

**Figure 4:**
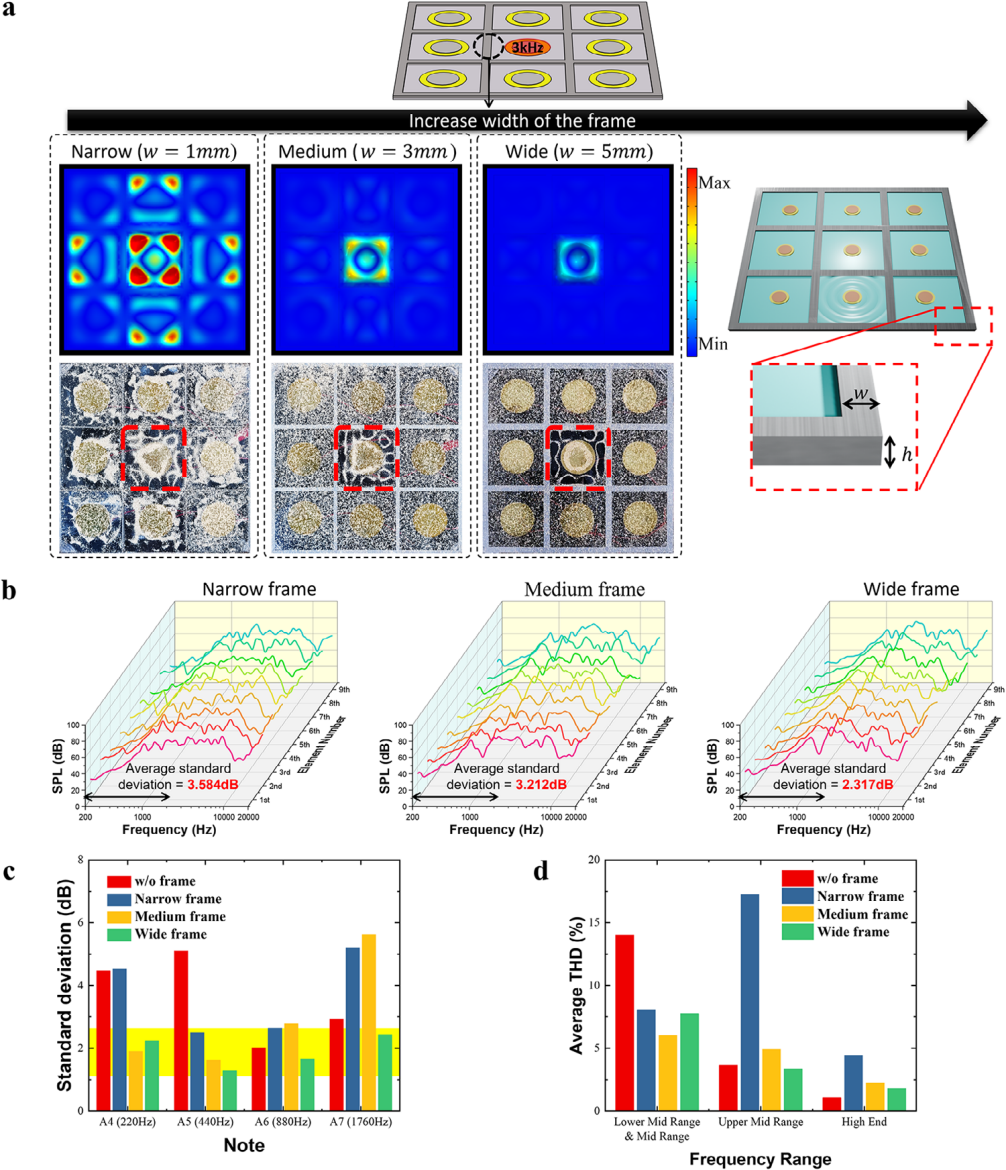
Speaker characteristics with varying frame widths. a) FEM results, and Chladni patterns illustrate the surface vibration of the diaphragm with varying frame widths that divide the regions of each exciter when a signal is applied to the central one. b) Frequency response of the panel speaker with varying frame widths dividing the regions of each element. c) SPL deviation of musical notes generated by 9 exciters in the piezoelectric elements array of the speaker. d) Average THD of sound generated by 9 exciters in the piezoelectric elements array of the speaker across the frequency bands of audio engineering.

**Figure** **4** illustrates the speaker characteristics with varying frame widths. The Chladni pattern, created using fine‐grain sand, along with the FEM results of surface vibrations on the diaphragm, were investigated when only the central piezoelectric element was excited at specific frequencies. (Figure 4a). When comparing the results of speakers with different frame widths, vibrations from the central exciter spread to neighboring regions in the narrow frame model (w = 1 mm). However, in the medium (w = 3 mm) and wide frame models (w = 5 mm), vibrations were confined to the exciter's designated area. Analysis of Chladni patterns and the simulation results across different frame dimensions and frequencies demonstrates that wider frame widths significantly improve vibration localization (Figures S3 and S4, Supporting Information).

Note that frame width also impacts the frequency response of each exciter in a 3×3 array configuration (Figure 4b). Figure S7a (Supporting Information) presents a plot overlaying the frequency responses of 9 exciters for comparison. Calculating the standard deviation in response between exciters in each speaker within the 200–2000 Hz range, where human hearing is most sensitive, reveals that the narrow, medium, and wide frame speaker models have standard deviations of 3.584, 3.212, and 2.317 dB, respectively. This indicates that wider frames reduce response deviation among exciters within this critical range. Comparing the SPL standard deviations of the nine exciters at frequencies corresponding to A notes reveals that the standard deviations are higher for A4 and A5 when the vibrations from the exciters are not confined (Figure 4c). If the frame was not sufficiently wide, a higher standard deviation was observed at A7. However, the wide‐frame speaker maintained a low standard deviation across these frequencies. The standard deviation data across frequencies is presented in Figure S7b (Supporting Information). In the 200–2000 Hz range, where human hearing is most sensitive, we observed that a wider frame width leads to lower deviation. However, beyond 2000 Hz, the influence of the frame became less significant, and at frequencies above 1 0000 Hz, the deviation tends to increase. We also measured THD and calculated average THD across different frequency ranges (Figure S7c, Supporting Information; Figure 4d). Models without confined vibrations exhibit high THD in the lower mid and mid‐range frequencies, while narrow and medium‐width frames show higher THD in the upper mid‐range and high frequencies. The wide frame model, however, consistently maintains low THD across all frequency ranges, indicating superior sound quality across the spectrum.

#### 2.3.2 Variation of Speaker Response with Frame Height

**Figure 5:**
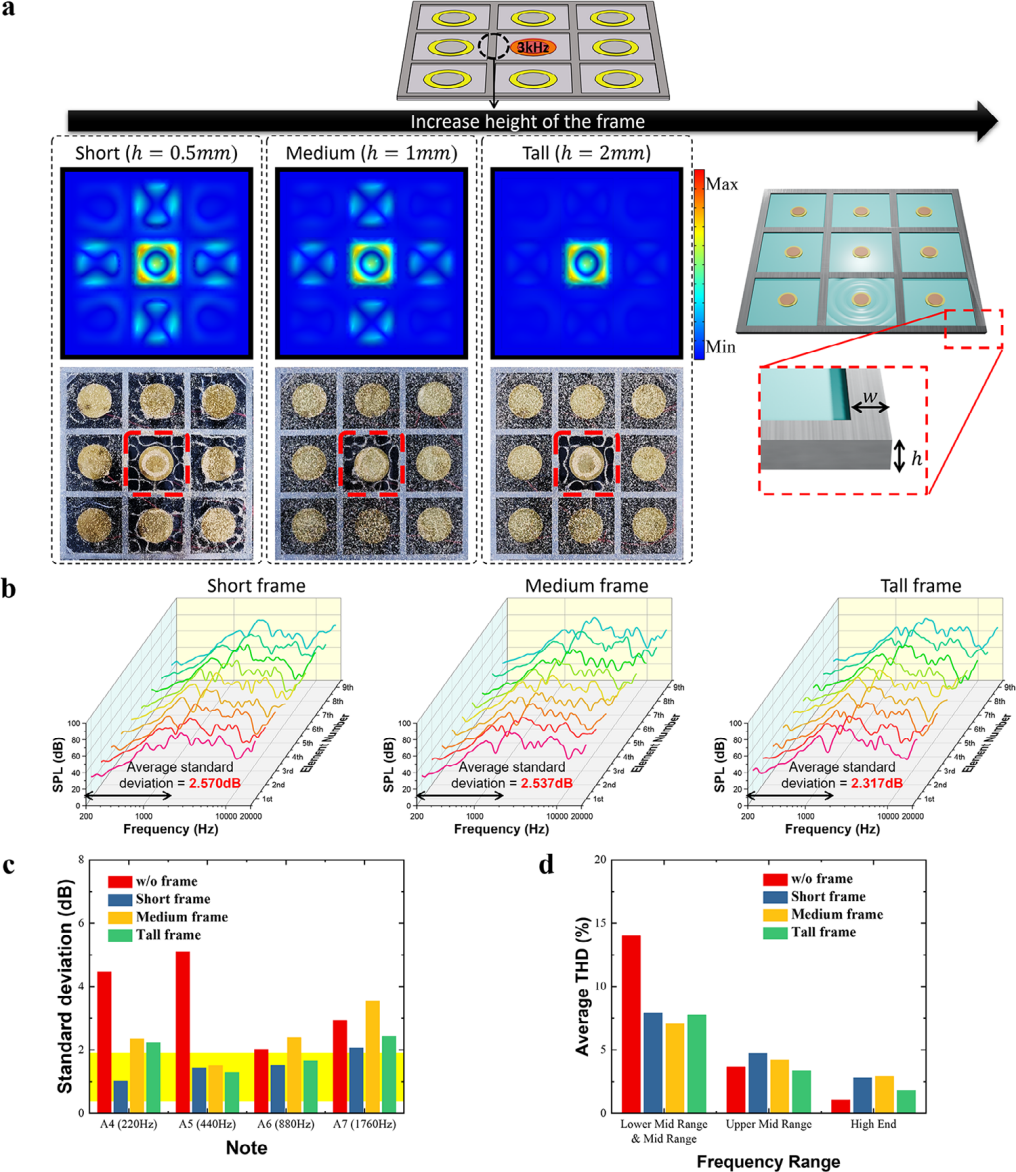
Speaker characteristics with varying frame heights. a) FEM results and Chladni patterns showing the surface vibration of the diaphragm with varying frame heights that segment each exciter's region when a signal is applied to the central exciter. b) Frequency response of the panel speaker with different frame heights. c) SPL deviation of A notes produced by 9 exciters in the piezoelectric element array of the speaker. d) Average THD of sound produced by 9 exciters in the piezoelectric element array of the speaker across the frequency bands used in audio engineering.

Vibrating sound waves generated by the exciter on the diaphragm propagate and are partially absorbed by materials with different stiffnesses. Sound waves absorbed by the frame are attenuated within the frame and then either transmitted back to the diaphragm or released into the air, generating sound. Therefore, we hypothesized that increasing frame height would enhance wave attenuation, leading to stronger vibration confinement. To verify this, we examined the variations in speaker characteristics at varying heights, keeping the frame width fixed (w = 5 mm). First, we applied a signal to the central exciter and examined the vibrations using FEM simulation and Chladni patterns (**Figure** **5**a). FEM sound simulation results clearly demonstrated that as the frame height decreases, the vibrations from the central exciter extend beyond the central region and propagate into the areas occupied by other exciters. Additionally, the Chladni patterns revealed that once the frame reaches a certain height, the vibrations no longer spread into neighboring regions. Additional experimentally measured Chladni patterns for varying frame heights (h = 0.5, 1, 2 mm) and frequencies are shown in Figure S3 (Supporting Information) and corresponding simulation results in Figure S4 (Supporting Information). Similar to Figure 5b, we measured the frequency response of the nine exciters in the speaker and calculated the deviation in the 200–2000 Hz range for each exciter's response (Figure 5b). The standard deviation was 2.570 dB for the short frame (h = 0.5 mm), 2.537 dB for the medium frame (h = 1 mm), and 2.317 dB for the tall frame (h = 2 mm), indicating that increased frame height leads to more consistent frequency responses across exciters (**Table** **1**). Figure S8a (Supporting Information) shows an overlay of the 9 frequency responses for comparison. When observing the SPL standard deviation of the nine exciters at frequencies corresponding to the A notes, it is evident that the standard deviation is higher for A4 and A5 in speakers where the vibrations of the exciters are not confined (Figure 5c). In contrast, frame confining vibrations exhibited consistently low standard deviation across different exciter positions, even without a clear trend in frequency response deviation by frame height. Although the standard deviation of frequency response across different frequencies does not show a clear trend with varying frame heights, it was observed that in the lower frequency range, dividing the regions between exciters with a frame resulted in a reduction in standard deviation (Figure S8b, Supporting Information). We calculated the average THD across different frequency bands for all exciters in the array, as shown in Figure 5d and Figure S8c (Supporting Information), from an audio engineering perspective. While no clear correlation was found between frame height and THD, the frame exhibited a noticeable reduction in THD at sensitive lower‐mid and mid‐range frequencies.

To demonstrate that this technology can be applied regardless of display size, we conducted additional FEM simulations, showing that the introduction of a frame effectively confines each exciter's vibration region, preventing crosstalk and ensuring consistent sound uniformity across arrays of different scales. Figure S9 (Supporting Information) presents the overlayed frequency responses and the average standard deviation in the 200–2000 Hz range for three different panel and exciter array sizes, comparing configurations with and without the frame. In all three cases, the use of a frame for vibration localization resulted in a lower deviation within the specified frequency range. In the 2×2 panel configuration, all exciters are positioned at the corners, and rotating the panel preserves their relative positioning. As a result, even without a frame, deviation remains low due to symmetrical exciter placement. Discrepancies between simulation and experimental results may be attributed to the adhesive layer used in the experiment. OCA (Optically Clear Adhesive), which has a lower acoustic impedance than PET, likely caused additional vibration attenuation, further enhancing localization and frequency response consistency.

Building on this, the effectiveness of this technique primarily depends on acoustic impedance mismatches between the diaphragm and frame, suggesting that there are no inherent acoustic limitations when scaling the design to larger or different display types. However, since this approach involves attaching a grid‐like frame structure, an increase in the number of grids may significantly add to the device's weight. To address this, a potential solution is to group exciters into localized sections instead of fully isolating each exciter, thereby reducing the total frame weight while still effectively minimizing crosstalk. This modular approach allows the method to be applied across various display sizes, including large‐format panels.

**Table 1:** Standard deviation of frequency response in the 200–2000 Hz range for various speaker models.

Model	Standard deviation [dB]
Without inner frame	3.160
PC 5 mm 2T frame	2.950
SUS 5 mm 2T frame	2.317
SUS 5 mm 1T frame	2.537
SUS 5 mm 0.5T frame	2.570
SUS 3 mm 2T frame	3.212
SUS 1 mm 2T frame	3.584

Additionally, the reliability of the fabricated speaker was evaluated (Figure S10, Supporting Information), especially by examining the time stability of the speaker with exciter regions divided by frames. Continuous use of a device with reliability, whether mechanical or electrical, can lead to changes in mechanical properties due to stress and variations in electrical properties due to temperature changes, which can affect the speaker's performance. To assess time stability, the frequency response of the speaker was measured once, then the device was kept in an operating state for 10 min, followed by a re‐measurement (Figure S10a, Supporting Information). The response remained nearly constant within measurement error, indicating stable performance over time. Figure S10b (Supporting Information) shows the sound pressure generated by a sinusoidal signal at various frequencies, confirming that sound pressure linearly increases with peak‐to‐peak voltage, consistent with acoustic principles. Figure S10c (Supporting Information) demonstrates the sound pressure measured at varying distances from the center of the speaker. Notably, it was observed that the sound pressure decreases as the distance increases, with the sound pressure halving as the distance doubles.

### 2.4 Localized OLED Panel Speaker from Different Signals to Multiple Exciters

**Figure 6:**
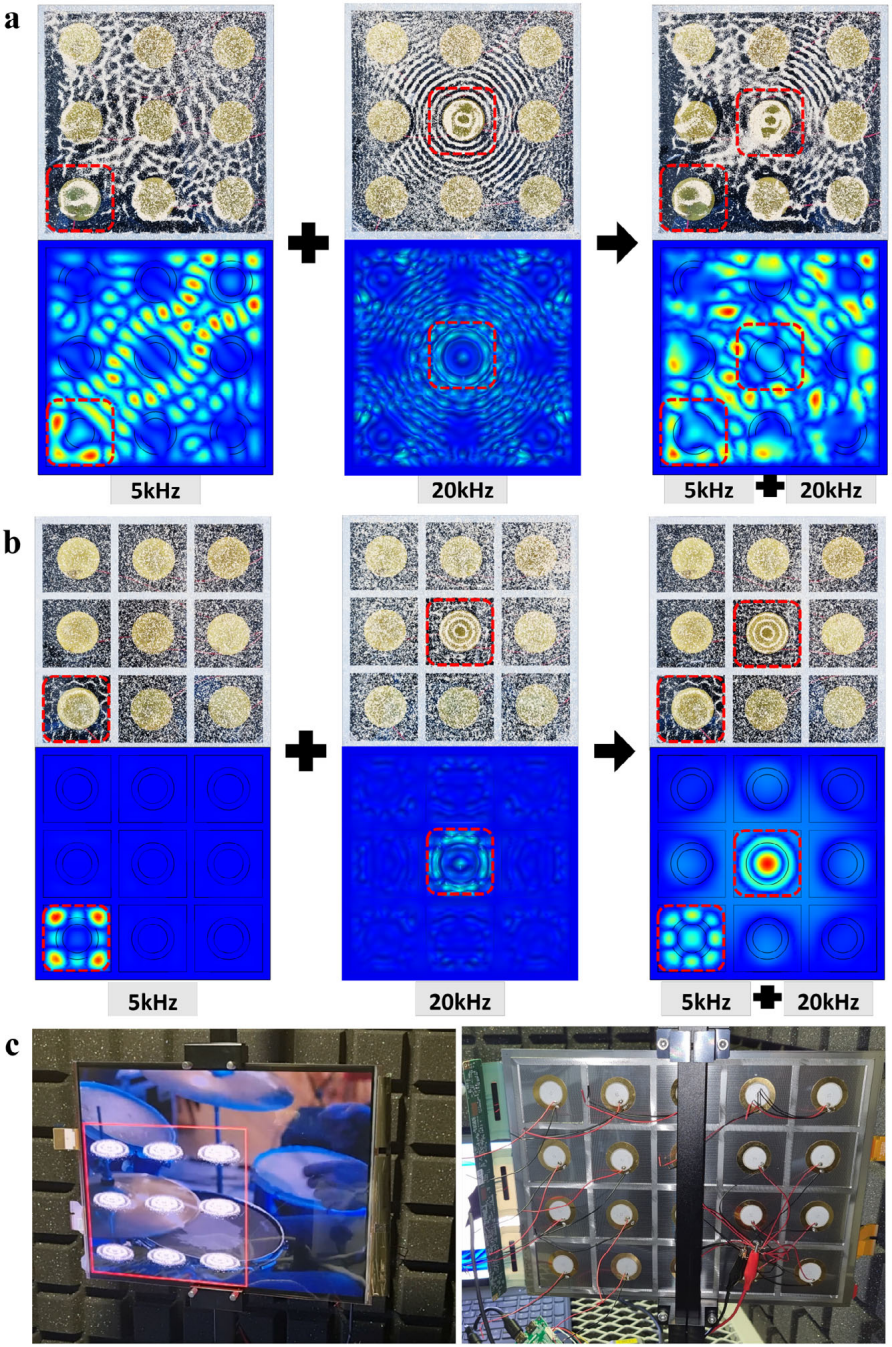
Chladni patterns and FEM results show the vibrations on a piezoelectric speaker's array of exciters when different frequencies are applied. a) Speaker with a frame only on the outer edges. b) Speaker where each region of the elements is separated by a frame. c) Piezoelectric panel speaker attached to OLED panel while playing songs.

When multiple exciters are placed on a single diaphragm and different sound signals are applied to each exciter in a stereo system, interference occurs between the vibrations generated by each exciter. However, by dividing the regions of each element with a frame, the vibrations are confined within their respective regions, preventing vibration interference. **Figure** **6**a,b shows the Chladni patterns and FEM results when two different frequencies are applied to two exciters. Without frame separation, applying a 5 kHz signal to the bottom‐left exciter and a 20 kHz signal to the center exciter results in overlapping vibration patterns, indicating interference (Figure 6a). However, with frame separation, the vibrations from the 5 and 20 kHz signals are found to be clearly isolated, demonstrating effective vibration separation (Figure 6b). Piezoelectric elements were attached to a single diaphragm panel (a 13‐inch OLED), enabling localized sound control, with sound played directly from the OLED (Video S2, Supporting Information). This experimentally confirmed sound crosstalk‐free, localized vibrating operation, synchronized with the visual display in a practical OLED display panel. Figure 6c shows the front and back views of the piezoelectric panel speaker attached to the OLED panel during audio playback. By overcoming the limitations of vibrating sound crosstalk that deteriorates speaker quality, we achieved improved sound crosstalk performance along with enhanced speaker functionality. Notably, no deterioration in image quality was observed; the quality remained consistent with that of a standalone OLED display.

## 3 Conclusion

In this study, we investigated localized sound emission synchronized with visual images in display panel speakers by examining crosstalk‐free diaphragm vibration and enhanced frequency response through localized control of multi‐arrayed piezoelectric exciters utilizing OLED's ultra‐thin vibration control for acoustic integration. Our primary goals were to minimize interference among exciters and achieve a uniform frequency response for precise sound localization. By introducing a vibration‐isolating frame structure optimized for shape, dimensions, and material properties, we achieved independently controllable crosstalk‐free sound emission in panel‐type piezoelectric speakers. The frames effectively confined surface vibrations to designated areas, preventing propagation to adjacent regions and enhancing frequency response uniformity, which reduced standard deviation and simplified the response compensation process. Furthermore, increasing the frame height and width improved frequency response uniformity and reduced Total Harmonic Distortion (THD) across a broader frequency range, resulting in clearer and more accurate sound reproduction. Frames with a large acoustic impedance contrast to the diaphragm material demonstrated superior performance in vibration confinement and overall speaker performance.

Consequently, we showcased a realistic 13‐inch OLED display with integrated localized sound, and long‐term reliability tests confirmed that the fabricated speakers maintained stable performance, showing consistent and predictable sound pressure level (SPL) variations with changes in input voltage and distance. These findings highlight the potential of frame‐based designs to substantially improve sound quality and durability in piezoelectric panel speakers and automotive applications. Beyond display‐integrated speakers, vibration localization has been widely explored in various fields,^[^ ^49^ ^]^ including thermodynamics for phonon transport control,^[^ ^50^ ^]^ energy harvesting systems such as Triboelectric Nanogenerators (TENGs) for efficiency enhancement,^[^ ^51^ ^]^ and acoustics for sound recognition using metamaterials.^[^ ^52^ ^]^ Additionally, spatiotemporal wave control plays a crucial role in chemical reaction compartmentalization,^[^ ^53^ ^]^ sensory signal processing,^[^ ^54^ ^]^ and audiovisual integration.^[^ ^7^ ^]^ In vehicle audio systems, panel speakers offer weight and space advantages over conventional electromagnetic speakers but suffer from crosstalk and vibration interference. Our frame‐based vibration localization mitigates these issues by confining vibrations within specific regions, ensuring precise sound localization. This method is particularly suited for dashboard‐integrated OLED speakers and multi‐zone in‐car sound systems, where spatially optimized audio is essential. These applications underscore the broad impact of vibration localization, suggesting its potential to advance not only display and audio technologies but also energy, thermal management, and chemical processing systems.

## 4 Experimental Section

### Fabrication of Piezoelectric Panel Speaker

A commercial PZT element was attached to a 100 µm thick PET film, which served as the diaphragm, using OCA. The PZT element had the following dimensions: a bottom electrode diameter of 27 mm and height of 0.14 mm, a PZT diameter of 18.6 mm and height of 0.23 mm, with a thin film top electrode deposited on its surface. Additionally, SUS 304 or PC, processed with a laser cutter, was also adhered to the PET film with OCA.

### Experimental Setup for Speaker Response

The frequency response and THD measurements were conducted inside an anechoic chamber (800 × 900 × 800 mm). Signal generation and data acquisition of the sound output from the speakers were controlled using LabVIEW. A PXIe‐5413 waveform generator produced a 10 V peak‐to‐peak sine wave, sweeping frequencies from 200 to 2 0000 Hz over 30 s, which was applied to the speaker elements. The sound output from the speakers was measured using a B&K 4966 microphone and a PXIe‐4494 sound and vibration module. To determine the range of vibrations generated by the piezoelectric exciter, Chladni patterns were measured using sand. A sine wave was applied to the centrally located element of the 3×3 arrays using a Tektronix AFG1022 function generator. Since the magnitude of the vibrations varied with frequency, the input voltage was adjusted accordingly to prevent excessive vibrations that could blow away all the sand from the diaphragm.

### FEM (Finite Element Method)

The diaphragm surface vibration analysis of the piezoelectric panel speaker was performed using COMSOL Multiphysics. The materials used in the simulations, such as copper, PZT, PET, PC, Aluminum, and Glass were sourced from the COMSOL Multiphysics material library. For SUS, a custom material was used with Young's modulus of 193 GPa, Poisson's ratio of 0.25, and density of 7900 kg m^−^^3^. The solid mechanics and electrostatics modules were used to apply signals to the piezoelectric elements, and the surface displacement was computed through frequency domain analysis. However, for Figure 6, where signals of different frequencies were applied to a single element, a time‐dependent analysis was used instead to compute the diaphragm surface vibration.

## Conflict of Interest

The authors declare no conflict of interest.

## Supporting information



Supporting Information

Supplemental Video 1

Supplemental Video 2

## Data Availability

The data that support the findings of this study are available from the corresponding author upon reasonable request.
