# Supplementary material for: Localized Sound‐Integrated Display Speaker Using Crosstalk‐Free Piezoelectric Vibration Array
Source: Adv Sci (Weinh). 2025 Apr 25;12(27):2414691. doi: 10.1002/advs.202414691 (PMC12279236; doi:10.1002/advs.202414691)
Supplement: Supplementary file 1 — Supporting Information [file ADVS-12-2414691-s002.docx]

Supporting Information

Localized Sound-Integrated Display Speaker Using Crosstalk-Free Piezoelectric Vibration Array

Inpyo Hong^1^, and Su Seok Choi^1,2^*

Graduate School of Semiconductor Technology and Department of Electrical Engineering

Pohang University of Science and Technology (POSTECH), Pohang, 37673, Korea

^1^I. Hong and Prof. S. S. Choi*

Graduate School of Semiconductor Technology

Pohang University of Science and Technology (POSTECH),

Pohang 37673, Republic of Korea

^2^Prof. S. S. Choi*

Department of Electrical Engineering

Pohang University of Science and Technology (POSTECH)

Pohang 37673, Republic of Korea

* Corrsponding author E-mail: [choiss@postech.ac.kr](mailto:choiss@postech.ac.kr)

**
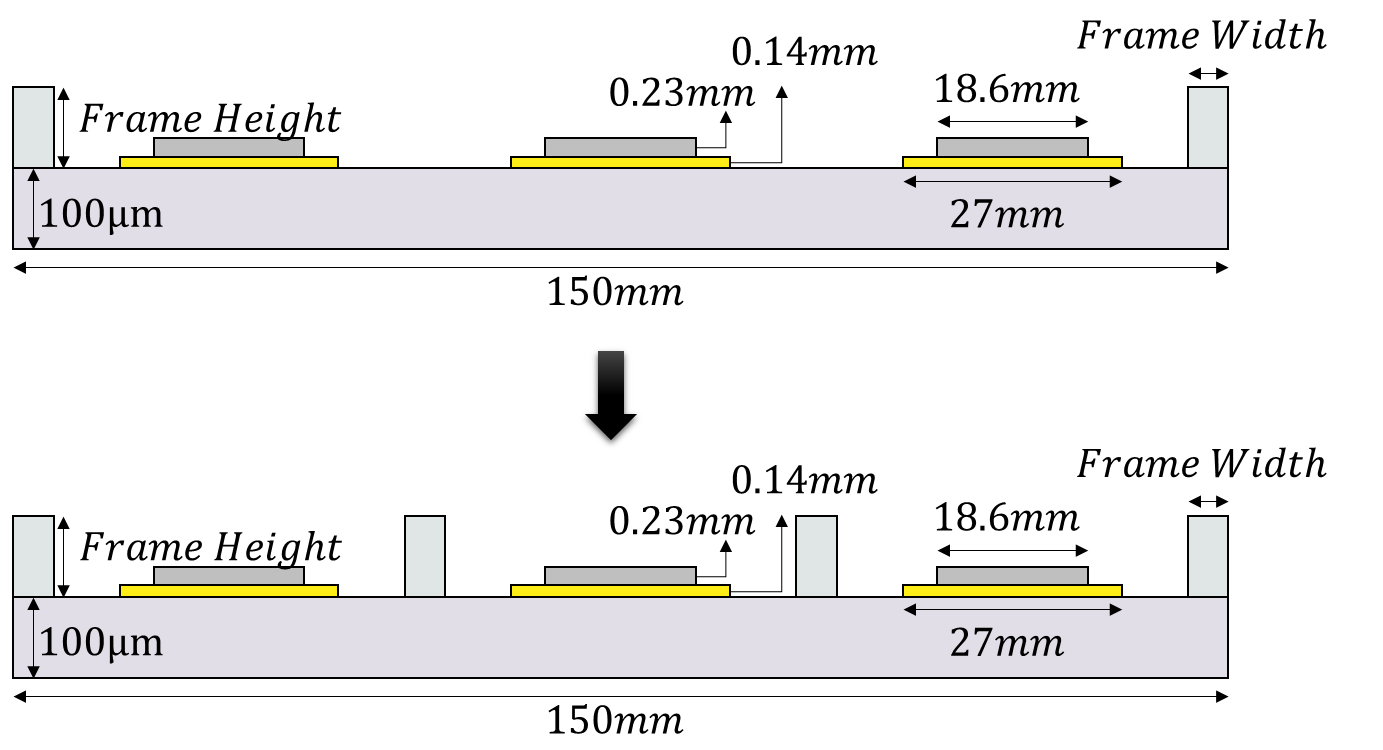
**

**Figure S1.** Detail dimension of the isolated speaker.


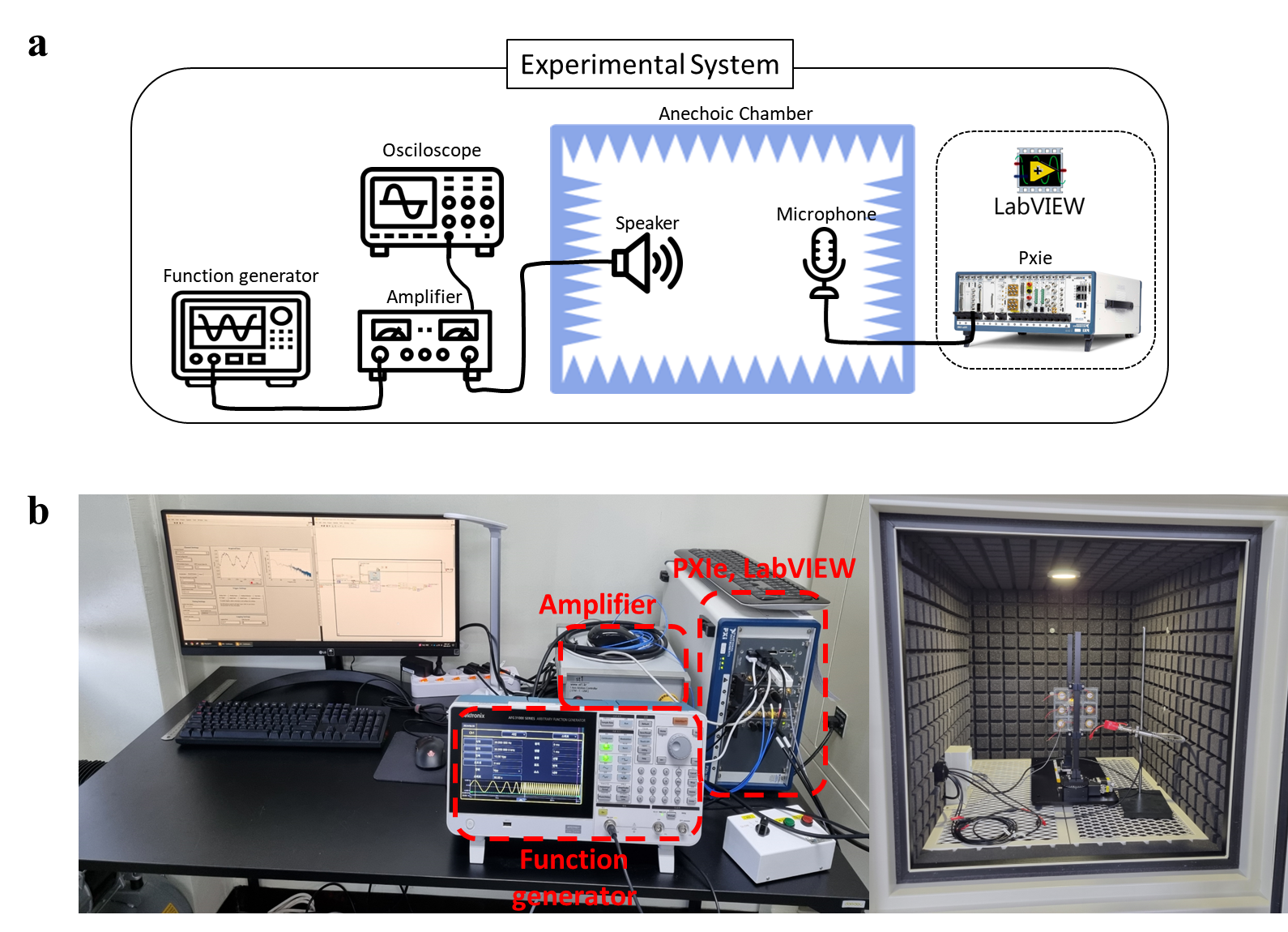


**Figure S2.** Experimental setup. **a,** Schematic of experimental system. **b,** Photograph of experimental system.


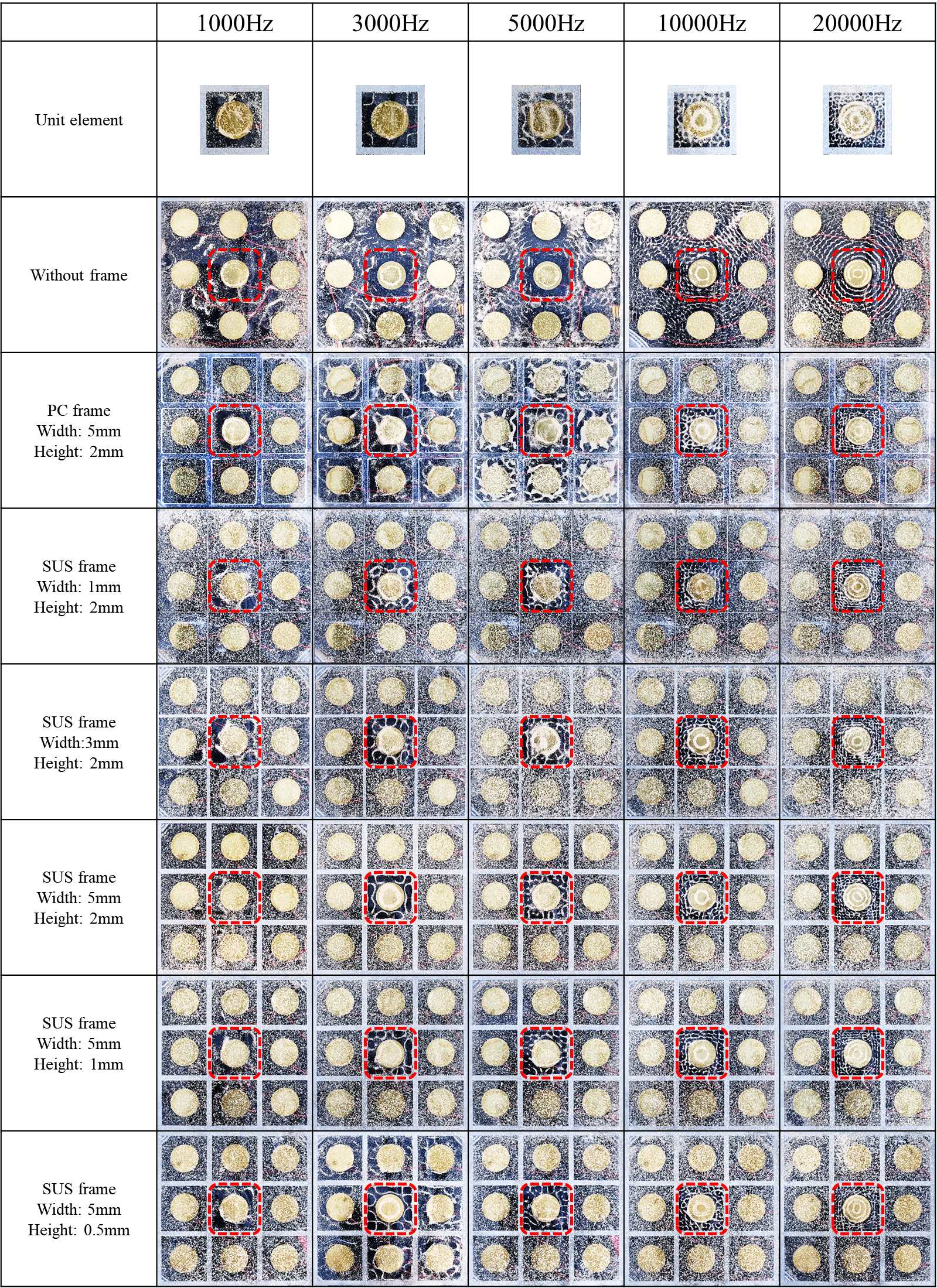


**Figure S3.** Chladni patterns at various frequencies for speakers with frames of different sizes.


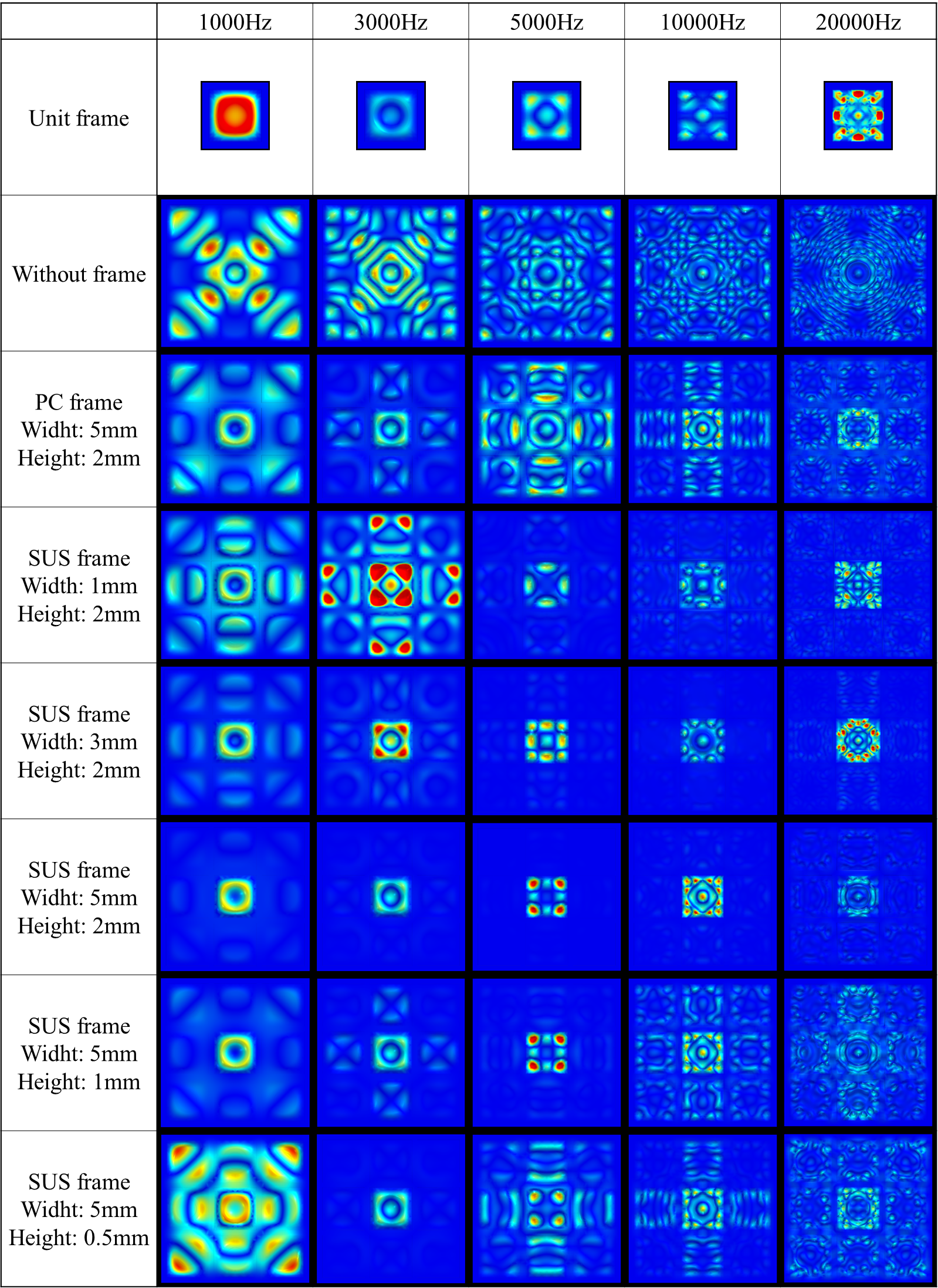


**Figure S4.** Surface displacement of the diaphragm at various frequencies, calculated using FEM, for speakers with frames of different sizes.

**
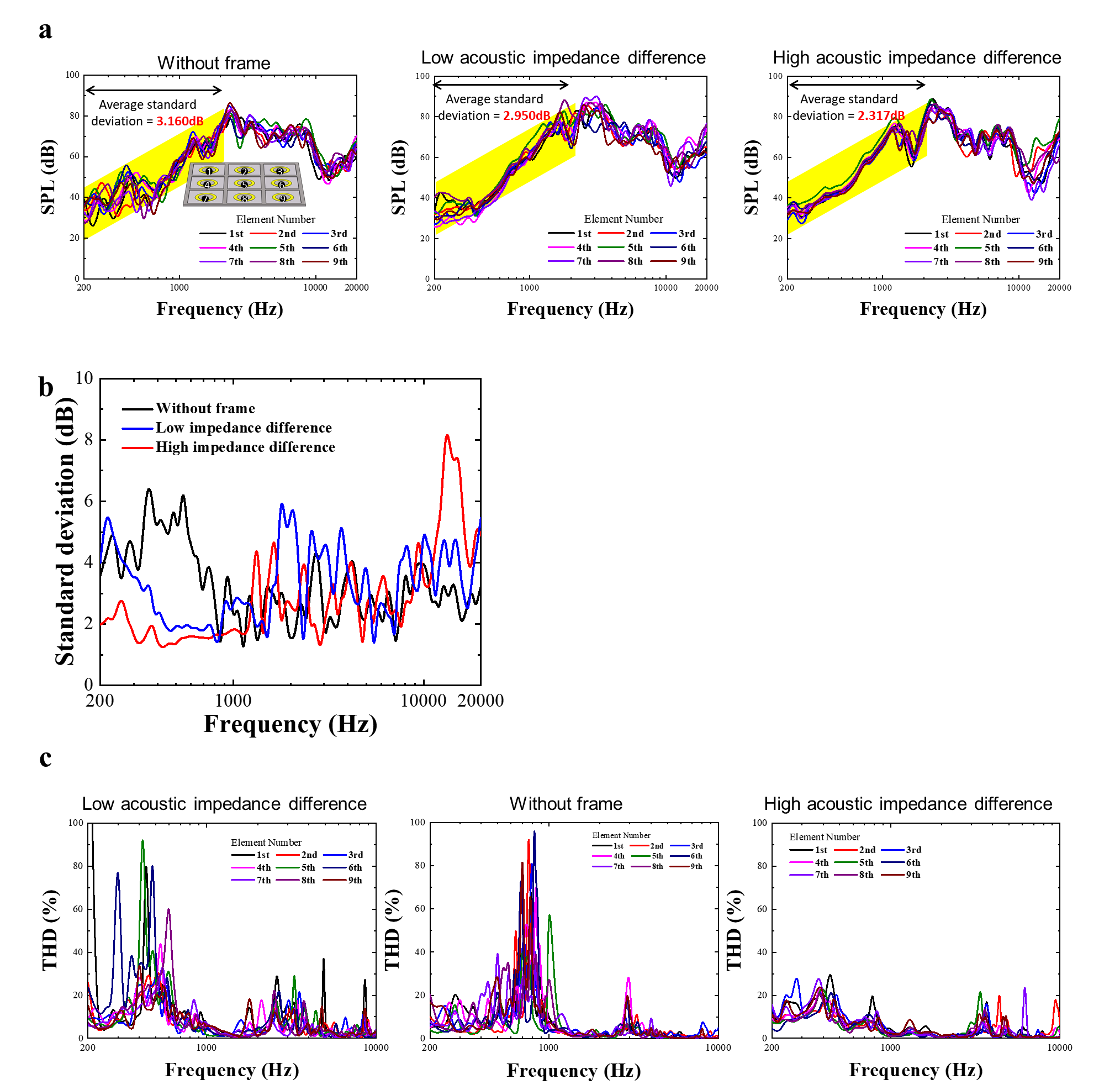
Figure S5.** Speaker responses of the piezoelectric panel speaker determined by the acoustic impedance difference. **a,** Frequency response of 9 elements. **b,** Standard deviation of the frequency response for the 9 exciters. **c,** THD measurement results.

**
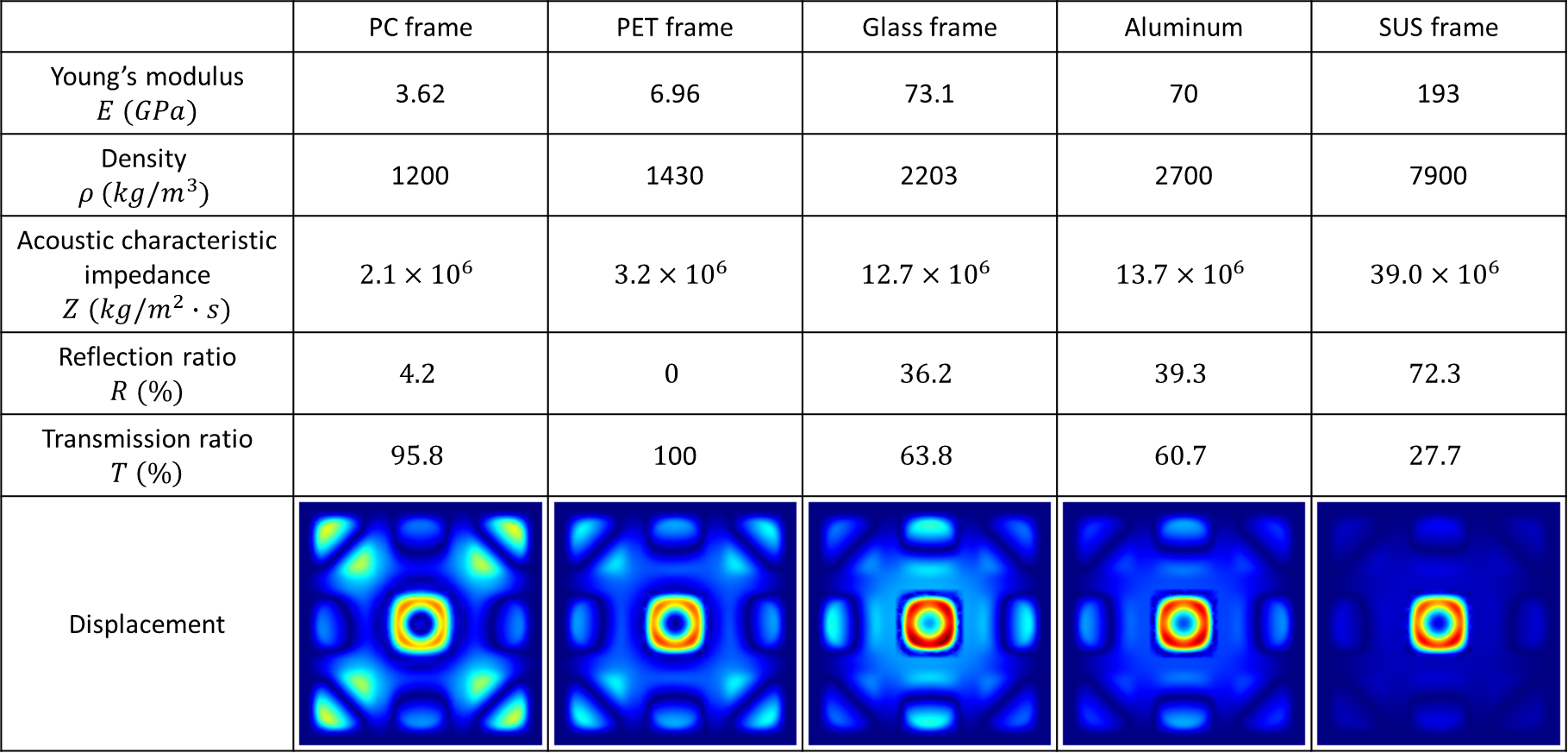
**

**Figure S6.** Diaphragm surface vibrations at 1000Hz and reflection and transmission ratios for various frame materials with different acoustic characteristic impedances in comparison to PET diaphragm.


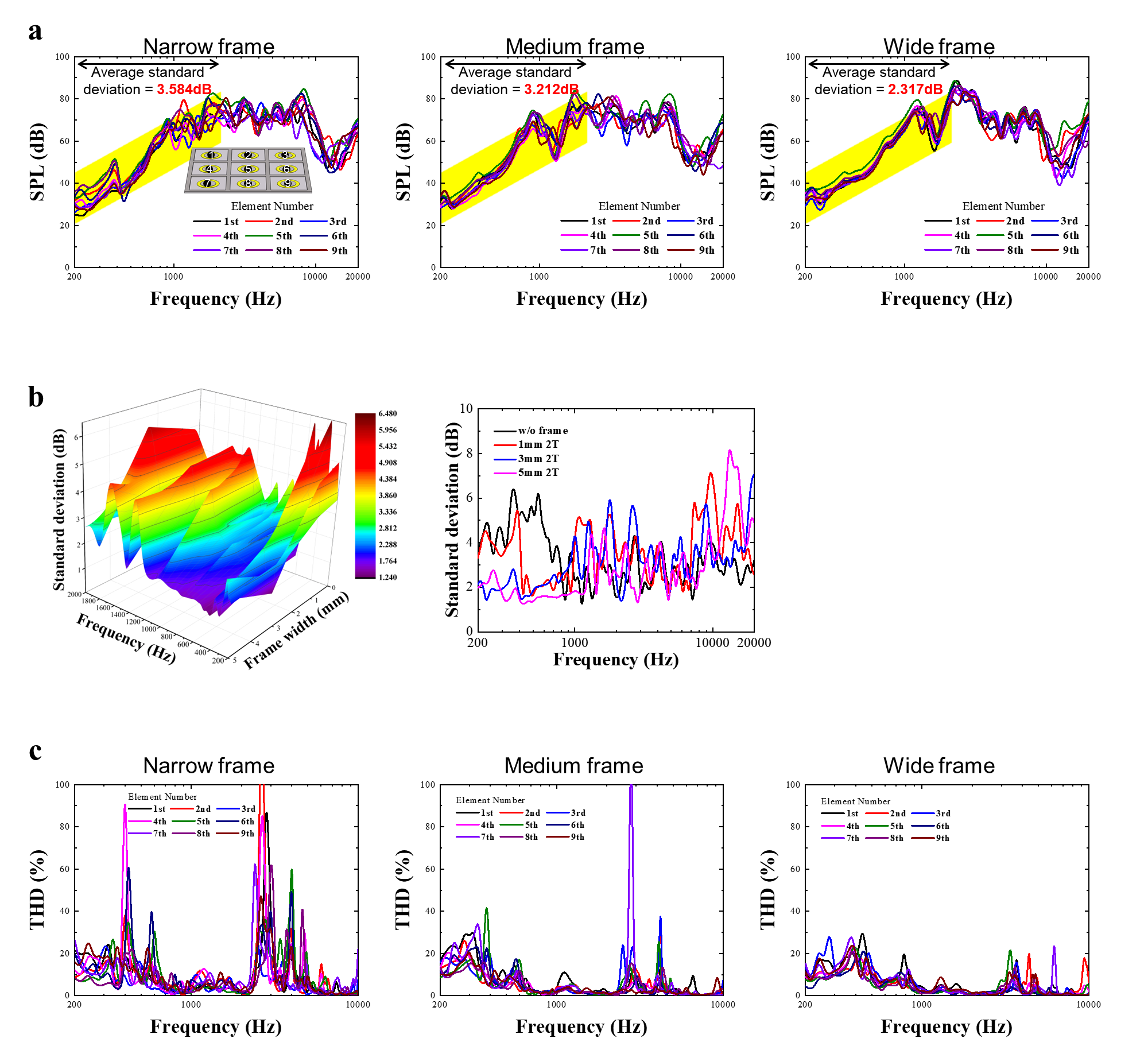


**Figure S7.** Speaker responses of the piezoelectric panel speaker with different frame width. **a,** Frequency response of 9 elements. **b,** Standard deviation of the frequency response for the 9 exciters. **c,** THD measurement results.


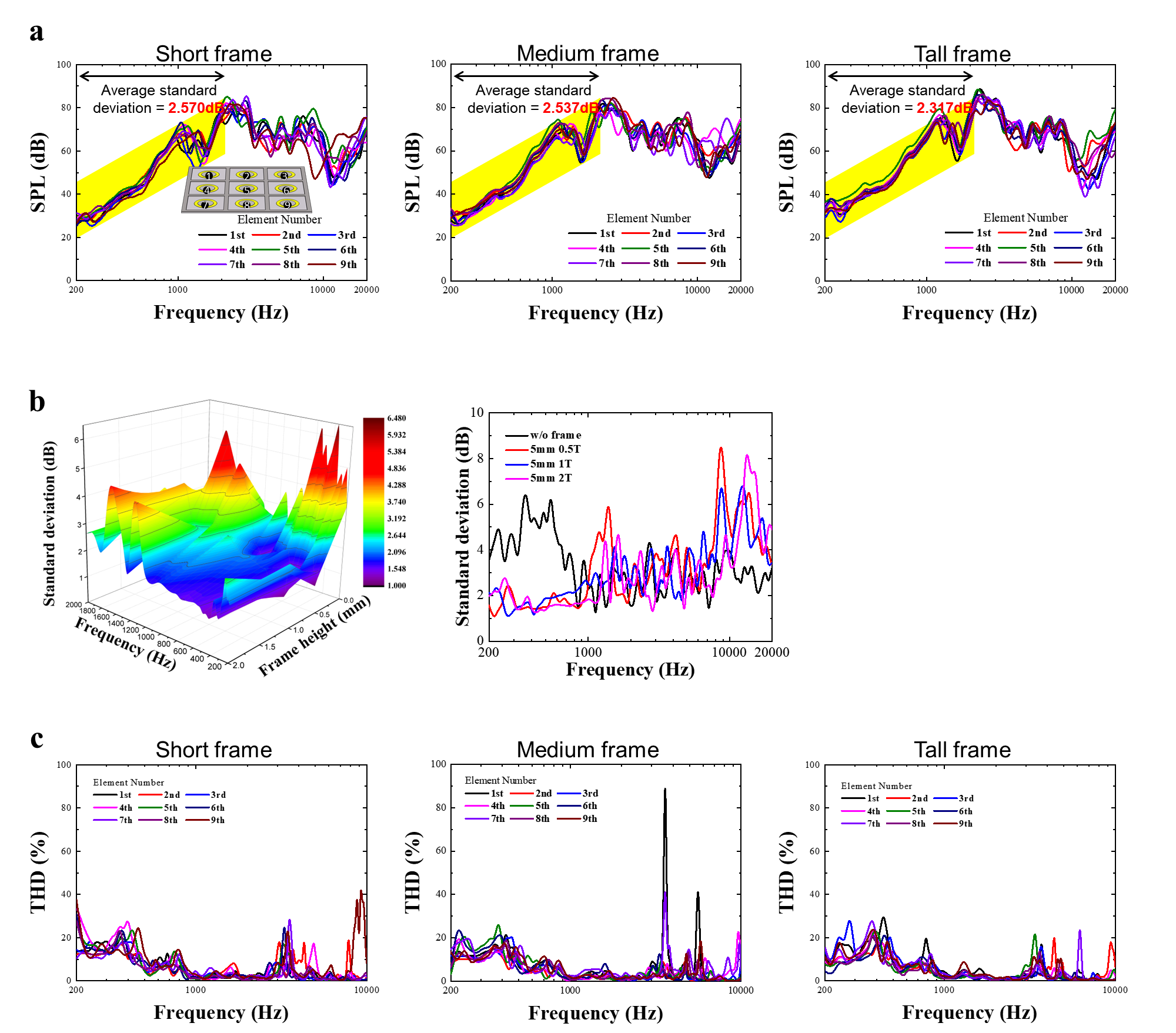


**Figure S8.** Speaker responses of the piezoelectric panel speaker with different frame height. **a,** Frequency response of 9 elements. **b,** Standard deviation of the frequency response for the 9 exciters. **c,** THD measurement results.


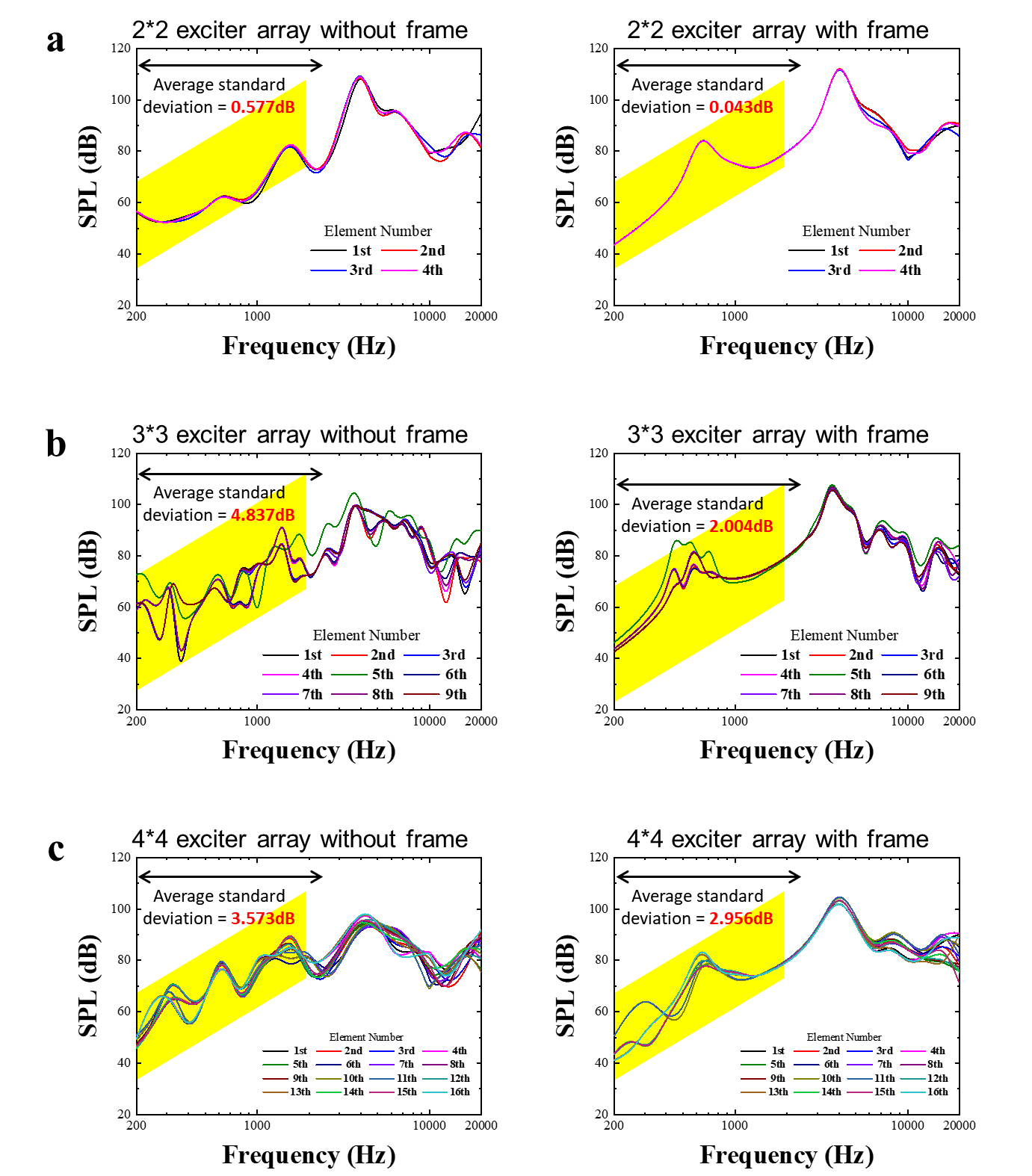


**Figure S9.** Overlayed frequency responses and average standard deviation in the 200~2000Hz range for three different panel and exciter array sizes, comparing cases with and without the frame. **a,** 2*2. **b,** 3*3. **c,** 4*4.


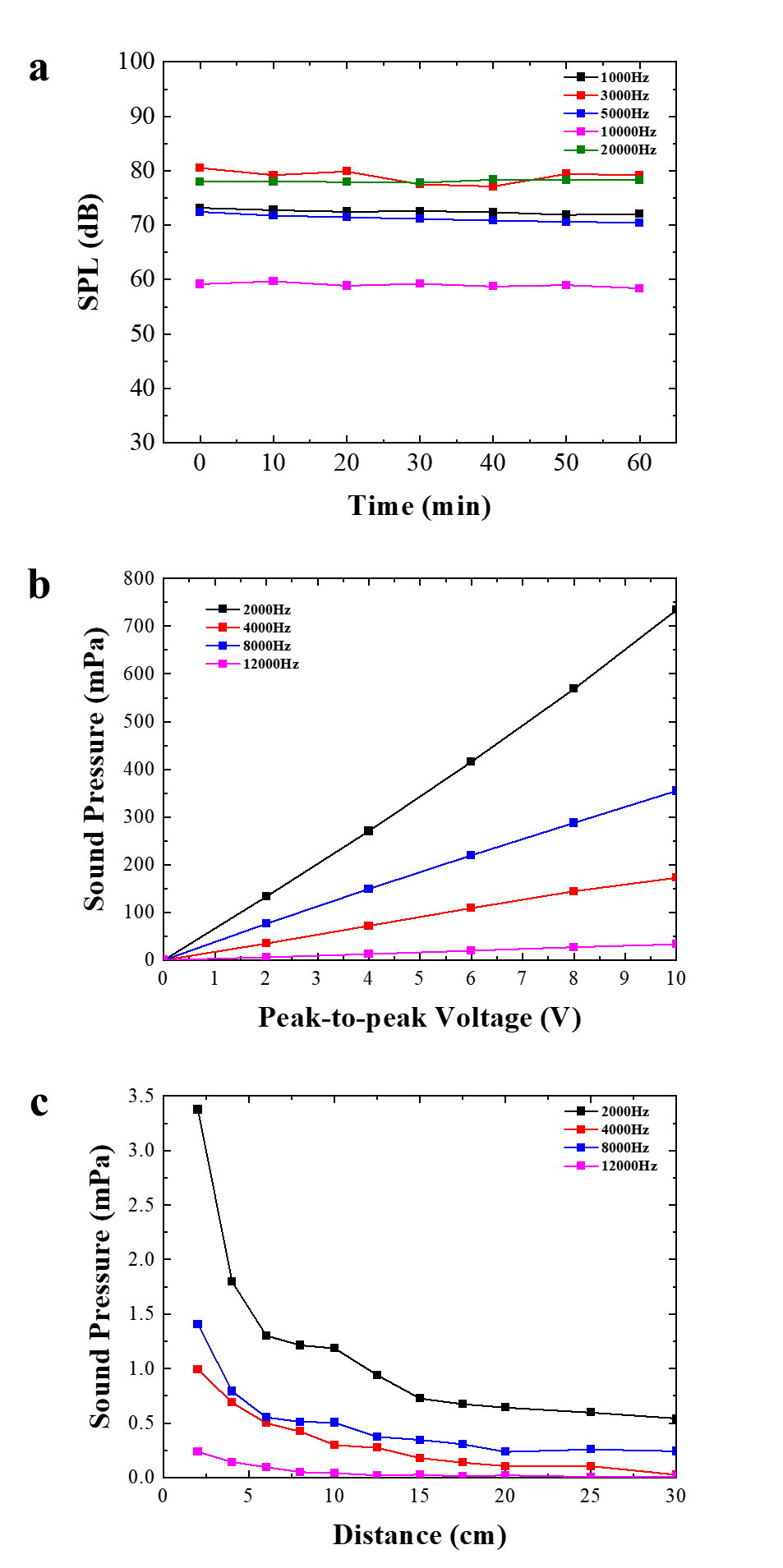


**Figure S10.** Speaker reliability tests. **a,** Time stability calculated as the difference in SPL between the initial state and 10 minutes of operation. **b,** The sound pressure showing linearity as a function of applied voltage. **c,** SPL for some frequencies varies with distance.

Supporting Videos

**Video S1**

Chladni patterns for three types of speakers

**Video S2**

Piezoelectric panel speaker attached on OLED panel playing song
